# The human crystallin gene families

**DOI:** 10.1186/1479-7364-6-26

**Published:** 2012-12-01

**Authors:** Graeme Wistow

**Affiliations:** 1 Section on Molecular Structure and Functional Genomics, National Eye Institute, Bg 6, Rm 106, National Institutes of Health, Bethesda, MD, 20892-0608, USA

**Keywords:** Crystallins, Lens, Cataract, Evolution, Pseudogene, Heat-shock, Enzymes

## Abstract

Crystallins are the abundant, long-lived proteins of the eye lens. The major human crystallins belong to two different superfamilies: the small heat-shock proteins (α-crystallins) and the βγ-crystallins. During evolution, other proteins have sometimes been recruited as crystallins to modify the properties of the lens. In the developing human lens, the enzyme betaine-homocysteine methyltransferase serves such a role. Evolutionary modification has also resulted in loss of expression of some human crystallin genes or of specific splice forms. Crystallin organization is essential for lens transparency and mutations; even minor changes to surface residues can cause cataract and loss of vision.

## Background

Crystallin is a functional term that originated as a description of the highly abundant soluble proteins of the ‘crystalline’ (clear) vertebrate eye lens [1,2]. Crystallins fill the hugely elongated, terminally differentiated fiber cells of the lens and must survive without turnover throughout life while maintaining transparency and the molecular organization required for the refractive properties of the lens. As such, the complement of crystallins in the lens has been peculiarly sensitive to evolutionary pressures and has shown remarkable adaptation in different vertebrate lineages [1]. As a result, some crystallins have very restricted distributions among species. However, there is a core set of three originally identified classes, the α-, β- and γ-crystallins, that must have arisen in early aquatic ancestors, are widespread among vertebrates, and account for most of the protein content of the human lens. These three classes were defined mainly by the sizes of oligomers they form, from very large α-crystallin multimers (on the order of 500 kDa), through dimer- to octamer-sized β-crystallins (approximately 45 to 180 kDa) to γ-crystallin monomers (20 kDa) [3]. Once sequence data were obtained, it became clear that the multimeric β- and monomeric γ-crystallins are in fact part of the same βγ-crystallin superfamily. Although crystallins were originally identified in the lens, they also have roles in other tissues and have superfamily relationships with other proteins. Indeed, throughout evolution, crystallins seem to have been recruited from existing proteins whose structure and other properties happened to suit them for the new role in lens [4].

By analogy to the lens, “crystallin” has also been used to describe abundant soluble proteins in the cornea, another transparent tissue [5-7]. However this review will focus only the human genes for proteins that are highly abundant in the lens and have crystallin as part of a *HUGO Gene Nomenclature Committee (*HGNC) designation or a widely used name.

A useful resource to search for expressed sequence tag (EST) data for crystallins (and other eye-expressed proteins) is NEIBank (http://neibank.nei.nih.gov) [8] and the accompanying eye-centric genome browser, EyeBrowse (http://eyebrowse.cit.nih.gov/) which displays expression and disease gene information for eye genes of human and several other species. Details of gene organization and splice patterns described in this review can be conveniently viewed using EyeBrowse or the original UCSC browser (http://genome.ucsc.edu/).

### *CRYAA* and *CRYAB*: genes for α-crystallins

**Figure 1:**
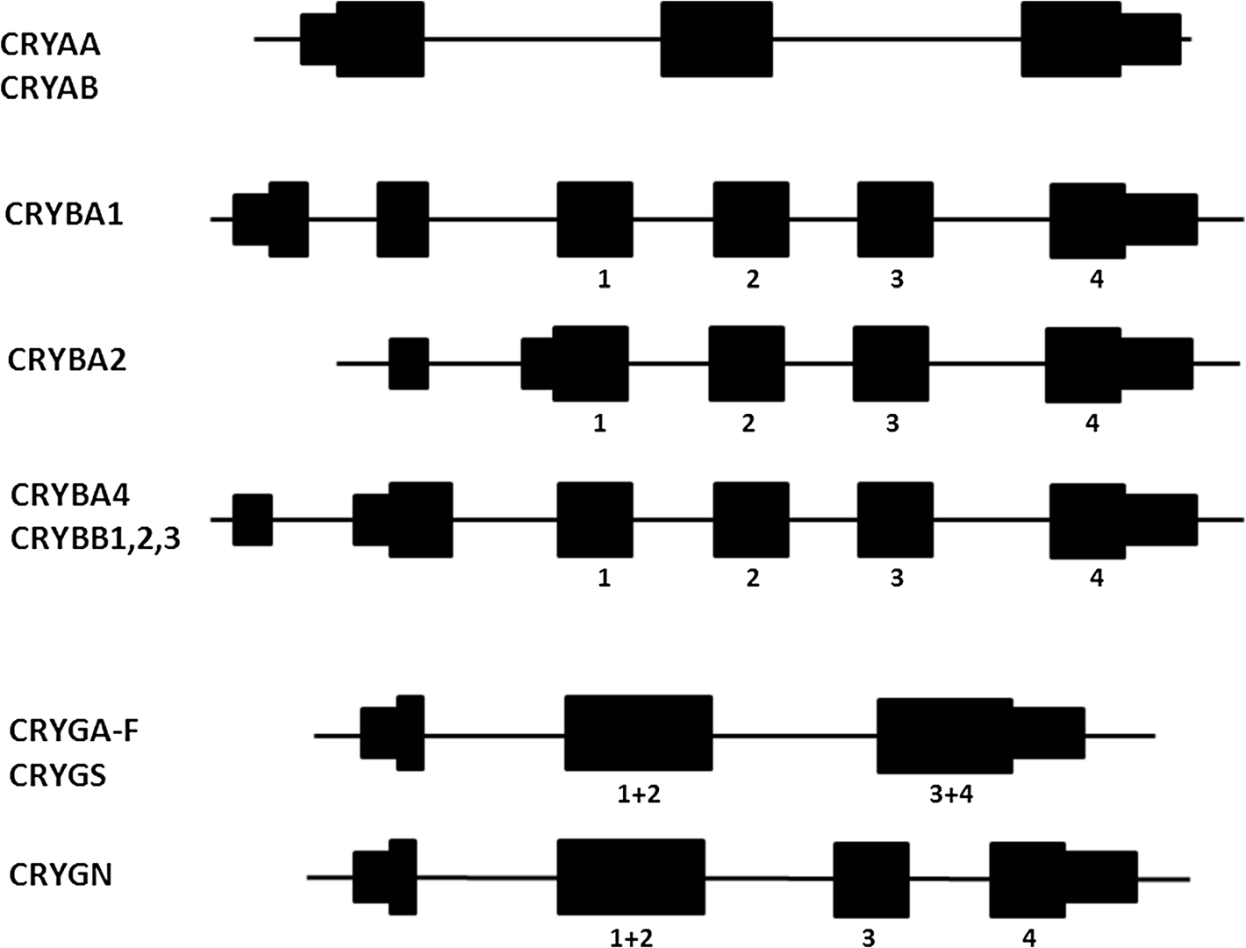
**Exon/intron structure of mammalian genes for α-, β- and γ-crystallins.** Exons are shown as boxes with ORF in thicker boxes. *CRYB* genes coding for β-crystallins vary in 5^′^-exon structure. The exons encoding the four structural motifs of the β-crystallin and γ-crystallin proteins are indicated by 1, 2, 3, and 4. Some mammals have an alternatively spliced exon in the first intron of *CRYAA*, but in humans this is a pseudoexon.

In humans (and apparently most terrestrial vertebrates) there are two α-crystallin genes, *CRYAA* and *CRYAB*, encoding αA- and αB-crystallins [2,9]. They are located in different chromosomes (21 and 11), but they are closely related in sequence and gene structure and are clearly the result of an ancient gene duplication. Both genes are 3 to 4 kb long with three exons (Figure 1). They have *open reading frames* (ORFs) of 173 (αA) and 175 codons (αB), corresponding to subunit sizes of approximately 20 kDa, and the two proteins are 54% identical in sequence. Ancestrally, the gene for αA- crystallin had three exons, and this is reflected in many species from fish to birds. However, during the evolution of the mammalian lineage, there was an innovation with the addition of an alternatively spliced exon, giving rise to a larger protein product (αAins) [10]. The ‘insert exon’ is retained in many species, particularly rodents. In primates, this alternative splice form was abandoned, but the remnants of the insert exon remain in human *CRYAA* as a pseudoexon [11]. While the sequence is no longer spliced into mature mRNA, it is largely intact, raising the interesting possibility that mutation could restore splicing and perhaps lead to a novel cataractogenic defect.

#### Superfamily relationships

**Figure 2:**
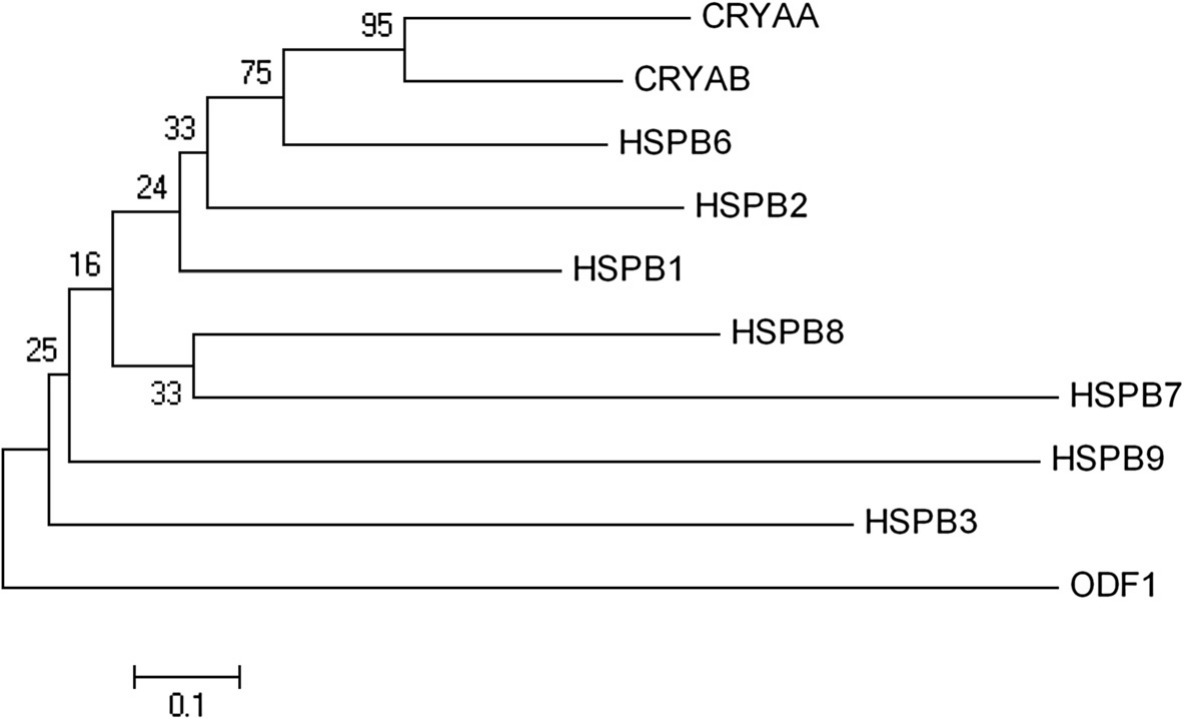
**Phylogenetic tree of the human sHSP gene family.** Sequences were extracted from the UCSC web browser. Translated ORFs were aligned and neighbor-joining trees were constructed using MEGA4 [15].

α-crystallins belong to the superfamily of small heat-shock proteins (sHSP) produced by even older duplications [12] (Figure 2). In humans, there are ten genes for sHSPs [13,14], and *CRYAA* and *CRYAB* also have the alternative systematic (but not widely used) names HSPB4 and 5. The tenth member has the HUGO name ODF1 [13]. Since the α-crystallins are the archetypes of the superfamily, the core-conserved region of the sequence for all these proteins is referred to as the α-crystallin domain [9]. Indeed, two of the non-crystallin members of this superfamily, *HSPB6* and *HSPB9*, have ‘alpha-crystallin-related’ as part of their HUGO designation. Although the two α-crystallin genes are not themselves linked, *CRYAB* is located head-to-head (adjacent 5^′^ regions, transcribed in opposite directions) with the *HSPB2* gene.

#### Functional implications

The relationship with sHSPs is not merely of evolutionary interest: αB-crystallin is a functional, stress-induced sHSP and is expressed in many tissues, notably muscle, sometimes at high levels (although not as high as in lens) [9]. In contrast, αA-crystallin is much more restricted to lens (although it has been found at low levels elsewhere, particularly in retina [16]) and may have evolved to specialize for the lens role, leaving the major sHSP role to αB-crystallin. This illustrates an evolutionary process in which a protein may acquire more than one role (for example, both sHSP and crystallin); if gene duplication then occurs, selection can lead to one or both copies of the gene specializing for one of the original functions [4].

A primary function of any crystallin is to contribute to the transparency and refractive power of the lens. However, other properties may also be important. In the case of the α-crystallins, the chaperone-like functions of the sHSPs have clear implications for roles in preventing aggregation of misfolded proteins with obvious benefits to lens transparency [9]. They may also have important interactions with other cellular components, including cytoskeleton, which is of major importance in the highly elongated lens fiber cells [17].

Since *CRYAB* is now well known as a functional sHSP, there has been a tendency to extend this functionality to other crystallins when they are found in unexpected places. It is important to recognize that only *CRYAA* and *CRYAB* belong to the sHSP superfamily, and that functional roles for other crystallins are only slowly emerging.

#### Cataractogenic mutations

Several cataract-causing mutations have been identified in human α-crystallins. For *CRYAA*, three dominant cataractogenic mutations are known which involve substitution of one of two surface arginine residues: R49C [18], R116C [19] and R116H [20]. Other mutants include a recessive nonsense mutation which essentially eliminates the ORF, W9X [21] and ΔY118 [22].

For *CRYAB*, some dominant mutations cause cataract which is also associated with myofibrillar myopathy, reflecting the role of the gene in muscle [9]. Three of these involve truncations of the ORF: Δ464CT [23], Q151X [23], and Δ60C [24], while one other, R120G [25], involves substitution of a surface arginine. In contrast, another deletion/truncation mutation, Δ450A, is only associated with posterior polar cataract [26]. Another congenital cataract in a Chinese study was identified with R69C [22].

### Genes for β- and γ-crystallins

#### Domains and exons

**Figure 3:**
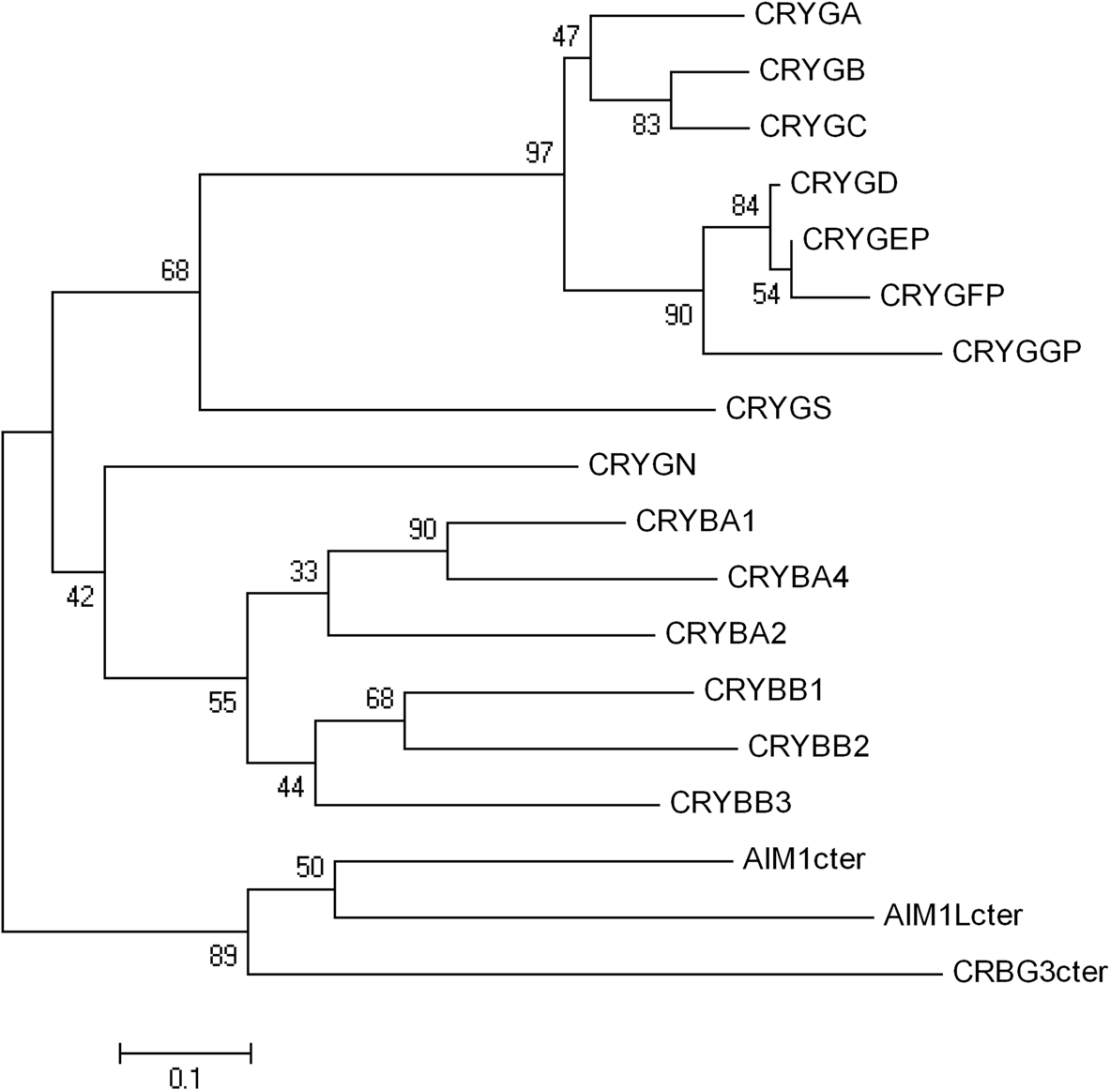
**Phylogenetic tree of the humanβγ-crystallin gene superfamily.** Sequences were extracted from the UCSC web browser. Translated ORFs were aligned and neighbor-joining trees were constructed as in Figure 2. *AIM1*, *AIML*, and *CRYBG3* contain internal repeats corresponding to three β-crystallin-like genes in addition to regions not related to crystallin genes. For simplicity, the third, most highly conserved crystallin repeat from each gene was used for this alignment (designated AIM1cter, etc.).

These two families are evolutionarily related but are also distinct (Figure 3). They share common core protein structures, with two similar domains, each composed of two characteristic-modified Greek key motifs. β-crystallins may also have N- and/or C-terminal extensions, exhibit greater structural flexibility in domain interactions, and form multimers, while γ-crystallins are strict monomers [1,2,27]. The two families also have distinct gene structures. In the genes for β-crystallins, each of the four structural motifs is encoded in a separate exon, while in (most) γ-crystallin genes, the ‘intra-domain’ introns are missing so that each domain is coded in a single exon (Figure 1). Genes for β-crystallin also have variable numbers of 5^′^ exons which account for N-terminal protein extensions and which may include a non-coding first exon.

### *CRYB* genes

**Figure 4:**
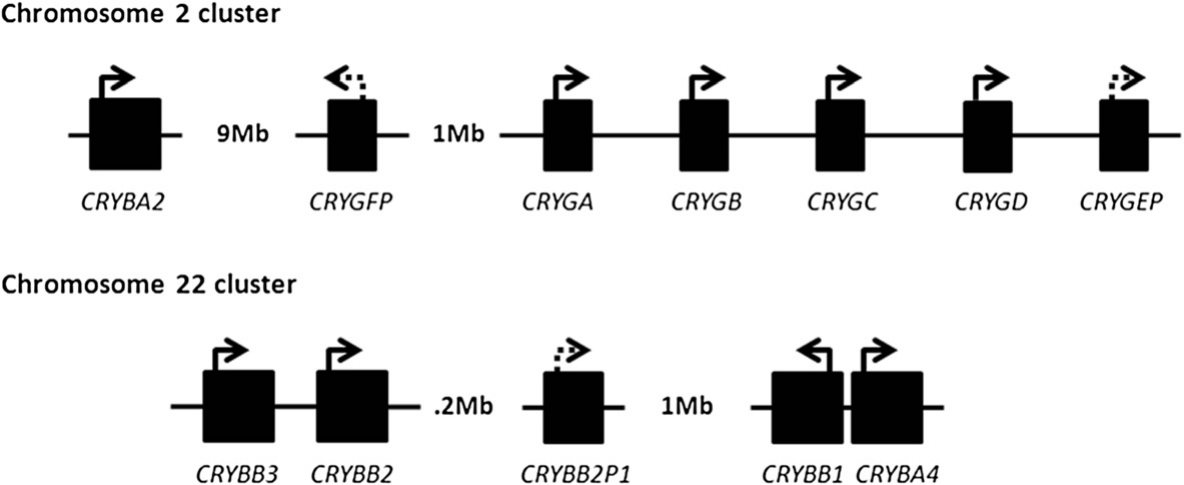
**Clustering of crystallin genes.** Gaps, indicated by approximate sizes, contain non-crystallin genes. Gene orientations are indicated with arrows (dotted for pseudogenes).

This multigene family has ancient origins in vertebrates, and indeed the six human genes have clear orthologs in fish [28]. β-crystallins are subdivided into acidic (A) and basic (B) subunits, encoded in *CRYBA*(*12,4*) and *CRYBB*(*123*) genes. Unlike the α-crystallins, four of the β-crystallin genes, arranged as two pairs, are close together on Chr 22 (Table 1, Figure 4).

**Table 1:** Human lens crystallin genes

**Crystallin genes**	**Chr**
α-crystallins	
***CRYAA***/**αA**	21q22.3
***CRYAB***/**αB**	11q23.1
β-crystallins	
***CRYBA1***/**βA1**, **βA3**	17q11.2
*CRYBA2*/βA2	2q35
***CRYBA4***/**βA4**	22q12.1
***CRYBB1***/**βB1**	22q12.1
***CRYBB2***/**βB2**	22q11.3
*CRYBB2 P1*	22q11.3
***CRYBB***3/**βB3**	22q11.23
γ-crystallins	
*CRYGA*/γ*A*	2q34
*CRYGB*/γ*B*	2q34
***CRYGC***/**γC**	2q33.3
***CRYGD***/**γD**	2q33.3
*CRYGEP*/γ*E*	2q33.3
*CRYGFP*/γ*F*	2q34
***CRYGS***/**γS**	3q27.3
*CRYGN*/γ*N*	7q36.1
*CRYGGP*	2p16.3
‘Enyzme-crystallin’	
***BHMT***/**ψ**	5q14.1

Gene/protein names and chromosome locations are shown. Genes/proteins in bold type are expressed at relatively high levels similar to orthologs in other mammals. Those in normal type seem to have reduced or no expression in human lens. Some are designated pseudogenes. The proposed crystallin designation for betaine-homocysteine methyltransferase (BHMT), ψ, does not imply pseudogene status.

#### CRYBA1

This single gene encodes two of the polypeptides that were originally identified by protein analysis of mammalian lenses, βA1- and βA3-crystallins, through the use of alternative ORF start sites [29]. *CRYBA1* is 7.6 kb long with six exons and is found on Chr 17, separated from other crystallin genes.

##### Cataractogenic mutations

Inherited cataracts are surprisingly frequently associated with splicing defects at end of exon 3 in this gene that probably lead to exon skipping. These are IVS3+1G>A [30], IVS3+1 G>C [31], IVS3+1 G>T [32], and IVS3+2 T>G [33] with similar examples found in other families. Another mutation (ΔG91) is the result of a 3-bp deletion of the codon for a highly conserved glycine required for normal protein folding [34]. Work in mice has revealed a role for βA3/A1-crystallin in retinal astrocyte function [35], but so far, it does not appear that astrocyte-related defects are associated with the human cataract mutations.

#### CRYBA2

This gene is also separated from other β-crystallin genes, being located on Chr 2, but is fairly close to a cluster of genes encoding γ-crystallins (Figure 4). The *CRYBA2* gene is 3.2 kb long and the major form has five exons. A variant transcript in which the first intron is included is also represented by several cDNA clones; however, since the ORF starts in exon 2, there is no difference in predicted protein product. No human cataract mutants have yet been identified in this gene. Although cDNAs from this gene are abundant in an adult human lens cDNA library [36] for some time, it seemed that no protein for βA2-crystallin was expressed in human lens (in contrast to bovine and rodent lens in which the orthologous protein was quite abundant. Subsequently, human βA2-crystallin was detected in the lens cortex but at relatively low levels [37], suggesting that translation of the mRNA may be inefficient.

#### CRYBA4

*CRYBA4* is part of a cluster of β-crystallin genes that spans about 1.5 Mbp on Chr 22. The general organization of this cluster is conserved in several species, including chicken. This raises the possibility that linkage is maintained for functional reasons, perhaps for coordinated expression, but this has not been determined. Indeed, *CRYBA4* is located head-to-head with *CRYBB1* (transcribed in opposite directions), a linkage which is found in chicken [38] and is even present in zebrafish (*Danio rerio*). *CRYBA4* has seven exons spread over 8.7 kb.

##### Cataractogenic mutations

Three coding sequence single-base transitions are associated with human cataracts. The F94S mutation gives rise to a dominant lamellar cataract [39]; L69P is associated with microphthalmia with cataract [39], and G64W is associated with congenital cataract and microcornea [40].

#### CRYBB1

The gene for βB1-crystallin is an 18.6 kb gene with 6 exons, a structure common to all three of the genes for βB-crystallins and *CRYBA4*.

##### Cataractogenic mutations

A variety of opacities and ocular development defects are associated with *CRYBB1*. Both dominant pulverulent (G220X) [41] and recessive nuclear (ΔG168) [42] cataracts have been associated with this gene. Autosomal dominant cataracts in Chinese families are associated with Q223X [43], R233H [44], and S228P [45]. Other mutants, X253R [46] and S129R [47], are associated with both congenital cataract and microcornea.

#### *CRYBB2* and *CRYBB2P1*

*CRYBB2* is a 12.2 kb, six-exon gene close to *CRYBB3* in the Chr 22 cluster. There is a partial pseudogene copy *CRYBB2P1* located about 250 kb away in the same cluster.

##### Cataractogenic mutations

A nonsense mutation (Q155X) associated with dominant cerulean (blue and white opacities) cataract has been identified in several different populations [48-51]. It has been proposed that this mutation results from independent gene conversion events between *CRYBB2* and *CRYBB2P1*[50,51]. However, the close similarity of gene and pseudogene sequences also raises the possibility of mispriming or the generation of chimeras in genomic PCR. A recent note describes a lack of specificity in primer sets used to identify *CRYBB2* mutants in another study [52,53], emphasizing the need for care in interpretation of results for this gene. Several other mutants have been reported including A2V (a surprisingly conservative change) [54], V146M and I21N [44], R188H [55] and S143F [56].

#### CRYBB3

*CRYBB3* is located about 25 kb 5^′^ to *CRYBB2* in the same orientation with no intervening genes. This linkage is conserved in chicken (where the separation is only about 2 kb), but not in zebrafish. The gene is 7.5 kb long with six exons.

##### Cataractogenic mutations

A recessive nuclear cataract is associated with a G165R mutation in *CRYBB3* that likely interferes with normal folding of the protein [57].

### Genes for γ-crystallins

In most mammals, there are eight genes for γ-crystallins [1,2,28,58]. Six encode a group of closely related proteins, γA-F, that are typically expressed early in development and therefore contribute mainly to the densest, central region of the lens, the lens nucleus. The other two members of the family, γS and γN, are more divergent in sequence, and their genes are located in different chromosomes (Table 1). All eight of these genes are expressed in the mouse lens; however, in humans, two of them are designated pseudogenes, another is effectively a pseudogene, and two others are expressed at relatively low levels in lens, suggesting that they too are in the process of evolutionary loss [28,59].

#### CRYGA-CRYGFP

These genes form a cluster in most mammals, and in the human genome, the orthologous genes and pseudogenes are clustered on Chr 2q (Figure 4). Unlike the β-crystallins, these γ-crystallins do not have orthologs in non-mammalian vertebrates; fish in particular have a large number of γM proteins that do not group with the mammalian proteins in phylogenetic analysis [28]. γ-crystallins appear to be highly specialized for the dense packing of the lens nucleus and for the creation of high refractive index, relatively inflexible lenses. This probably explains the trend for loss of γ-crystallins in the accommodating lenses of primates, in birds, and in other species such as guinea pig (*Cavia porcellus*) [60].

The genes all have three exons (Figure 1) and are typically 2 to 4 kb in length. The cluster clearly arose by gene duplication. In humans, *CRYGEP* and *CRYGFP* are pseudogenes with no evidence of expression [57]. For this reason, they are not represented as genes in human genome builds or on most browsers. This is unfortunate since the genes are still largely intact and have at least the potential for involvement in gene conversion with active genes or even reactivation. Indeed, reactivation of *CRYGEP* was at one time considered as the basis for a human cataract, although this later proved not to be the case [61]. Five of the clustered *CRYG* genes are arranged sequentially: *CRYGA*, *B*, *C*, *D*, and *EP*, within about 40 kb in the human genome (Figure 4). *CRYGFP*, despite its close similarity to *CRYGEP*, lies about 1 Mbp 5^′^ to *CRYGA*.

cDNA and proteomics analyses show that most of the transcripts and proteins arising from this cluster in humans come only from *CRYGC* and *CRYGD*, with the latter being predominant [36,62,63]. This is reflected in known cataract mutations in γ-crystallin genes.

##### Cataractogenic mutations

In *CRYGC*, two mutations leading to single amino acid substitutions of surface residues, T5P [58] and R168P [64], and a frame-shifting 5 bp duplication in exon 2 (corresponding to the N-terminal domain of the protein) [65], all cause cataract. In *CRYGD*, five mutations leading to single amino acid substitutions, R14C [66], P23T [64,67], P23S [68], A35P [22], R36S [69], and R58H [58] and another causing truncation of the protein, W156X [64], give rise to several different forms of cataract.

#### CRYGS

This gene shares the three-exon structure of other γ-crystallin encoding genes; although at 6 kb, it is somewhat larger than typical. Indeed, the longer, first intron of the human gene appears to contain a transcriptionally active region, of unknown function, and rare, probably non-functional, alternatively splice products of the gene that incorporate part of this sequence have been noted [70]. In contrast to the genes of the Chr 2 cluster, *CRYGS* is well-conserved in evolution and is expressed at high levels in human lens, particularly in adult in the cortical region [36,70].

##### Cataractogenic mutations

The distribution of expression in the lens is consistent with the progressive polymorphic cortical cataract caused by the G18V substitution of a key residue of the Greek-key motif [71]. Another mutant, D25G has also been reported [22].

#### CRYGN

This is another gene with orthologs throughout vertebrates [28]. Its gene structure resembles an evolutionary intermediate between those that code for the β- and γ-crystallins, with the N-terminal domain of the protein coded in one uninterrupted exon, as in a γ-crystallin gene, and the two motifs of the C-terminal domain encoded in separate exons, as in a β-crystallin gene (Figure 1). The gene is expressed at low levels in mouse lens and other parts of the eye [28], but human *CRYGN* appears to be non-functional. No cDNAs with the expected splice pattern for a full ORF have ever been detected, although some rare transcripts, apparently both sense and antisense, have been observed in different, non-lens cDNA libraries. These may relate to the presence of a microRNA gene, miR-3907, in the third intron of the gene. Human *CRYGN* is probably a pseudogene that has been deactivated relatively recently in evolution. However, the gene is represented in builds of the human genome and even has a RefSeq entry. The gene is 10 kb long and is located on Chr 7.

#### CYRGGP

In addition to the full-sized genes for γ-crystallins with orthologs in other mammals, the human genome contains an isolated fragment of sequence similar to that encoding γD motif 2 in *CRYGD*, apparently associated with a line located on Chr 2p [59]. This has been given a HUGO designation as *CRYGGP*, but it is not represented in most genome browsers. The name is problematic as it suggests that, by analogy to *CRYGEP* and *CRYGFP*, it is a formerly functional gene that has been silenced in the human lineage, whereas it appears to be merely a translocated remnant.

##### Superfamily relationships

The βγ-crystallin superfamily contains many members in prokaryotes and eukaryotes. In the human genome, the major non-crystallin members of the superfamily are absent in melanoma 1 (*AIM1*) (also known as *CRYBG1*, for ‘βγ crystallin domain containing 1’), the related AIM1-like protein (*AIMIL* or *CRYBG2*) and *CRYBG3*. The proteins contain six βγ-crystallin-like domains encoded in exons arranged like those in β-crystallin genes [72,73]. Since the proteins encoded by these genes do not serve as crystallins, using the term to describe them is potentially confusing, at least from a functional point of view and the current nomenclature is inconsistent. The names *AIM1*, *AIML1*, and *AIML2* would better describe their family relationship. However, there is potential confusion here too. The acronym ‘AIM1’ has been used for different genes (*SLC45A2*, *AURKB*), while *AIM2* is the HGNC approved designation for another gene unrelated to *AIM1*.

*AIM1* spans almost 60 kb on human Chr 6. It consists of 20 exons, 12 of which code for βγ-crystallin motifs, corresponding to six domains possibly arranged effectively as a β-crystallin trimer [72]. Additional short exons in this region code for linkers between the two-domain β-crystallin regions. This region must have arisen by successive duplication of an ancestral, β-crystallin-like gene. The AIM1 protein also contains a long, proline-rich N-terminal region (over 1,000 residues) with no clear structural relationships and a C-terminal ricin-like trefoil domain. Interestingly, another connection between βγ domains and a trefoil domain has been observed in a protein discovered in skin secretions of a frog (*Bombina maxima*). The so-called βγ-CAT [74] is a dimer of a βγ-crystallin superfamily member and a trefoil domain protein. Although AIM1 is not a crystallin, it is expressed at moderately high levels in a human cornea cDNA library [75].

The *AIM1L* gene covers 32 kb on Chr 1, and *CRYBG3* covers 123 kb on Chr 3. Both these genes have similar exon/domain structures to AIM1, although *CRYBG3* appears to have two additional coding 5^′^ exons.

##### Functional implications

Members of the βγ-crystallin superfamily are quite diverse, and no single functional role is clear. However, some of them seem to be associated with cellular architecture. AIM1 expression is associated with changes in appearance and reduction of malignancy in melanoma cells [76], while another relative, EDSP/Ep36, is associated with plasma membrane in developing *Cynops* amphibian embryos [77,78]. Recently, it has been shown that mouse γS-crystallin associates with F-actin in the lens and can stabilize F-actin *in vitro*[79]. While not reaching the level of chaperone activity, it is possible that the characteristic βγ-crystallin protein domain has a generalized role in shepherding large oligomers (like cytoskeleton and other crystallins) and preventing aggregation.

### *BHMT* and other ‘enzyme crystallins’

As described above, some major crystallins are more or less specialized for their role in lens and may have no role, or at least only a minor role, in other tissues. However, during evolution, the molecular composition of the lens has adapted in response to changing environmental demands. In the human lineage, this has involved the loss of γ-crystallins, the loss of the alternative ‘insert’ exon of *CRYAA*, and (probably) decreased expression of *CRYBA2*, while genes for other non-crystallin proteins, such as *LGSN*/lengsin (exon loss) [80] and *LIM2*/MP19 (exon extension) [36], have also been affected. In many species, particularly when expression of γ-crystallins has been reduced, existing, stable proteins with the needed properties have been recruited to a new role as a crystallin through increased expression in lens. These are usually enzymes, so that, for example, in many birds and crocodiles the enzyme lactate dehydrogenase B became ε-crystallin in the lens while retaining its expression and function as an enzyme [81]. The same process has occurred in primates. Probably to compensate for, or to replace absent or down-regulated γ-crystallins, the developing primate lens expresses high levels of the enzyme BHMT [82]. In a human fetal lens cDNA library, *BHMT* is the most abundantly expressed gene (http://neibank.nei.nih.gov/cgi-bin/showDataTable.cgi?lib=NbLib0012). Thus, *BHMT* should certainly qualify as a human crystallin gene; however, the proposed name ψ-crystallin [82] has not been widely used perhaps because of the potential confusion with the symbol for pseudogene. So far, no genetic cataract has been associated with *BHMT*/ψ-crystallin, but it is certainly a candidate worth considering for congenital nuclear cataract, possibly microphthalmia. BHMT, as expressed in many tissues, is an eight exon, 20-kb gene on Chr 5 separated by about 20 kb from the related *BHMT2*.

The term crystallin may also arise in connection with other human genes whose orthologs in other species serve as crystallins. *CRYM* is the human ortholog of the gene that encodes μ-crystallin in many marsupials [83-85]. In humans, *CRYM* is expressed quite abundantly in retina and inner ear, where mutants are associated with deafness [86], and cDNAs for this gene are found in libraries from retina and fetal eye; however, it does not rise to ‘crystallin-levels’ of expression in lens. μ-crystallin belongs to an enzyme superfamily and has been identified as a ketamine reductase and also as a thyroid-hormone-binding protein [84,87,88].

*CRYZ* is the human ortholog of the gene that codes for ζ-crystallin in hystricomorph rodents (such as guinea pig) and camels [89]. The protein is a quinone oxidoreductase [90]which is not expressed at high levels in human lens, although it is moderately abundant in a human cornea cDNA library [75]. A related gene is designated as *CRYZL1*, and there is also a pseudogene copy, *CRYZP1*.

*CRYL* is the human ortholog of the gene that codes for λ-crystallin in lagomorphs (rabbits, hares, pikas) [91]. λ-crystallin is related to hydroxyacyl coenzyme A dehydrogenases but does not serve as a crystallin in humans.

The enzyme α-enolase (*ENO1*) is also sometimes referred to as τ-crystallin (see OMIM: *172430) because of its role as a crystallin in several non-mammalian species [92], but, although quite abundant as an enzyme in lens, it does not pass the (rather subjective) test to qualify as a human crystallin.

### Lens-preferred gene expression

Although proteins of very different superfamilies act as crystallins in the lens, they all acquired this role through increased gene expression in the lens. Some crystallins are essentially tissue-specific, most have some level of expression in other parts of the eye, while others have a completely separate machinery of expression in non-ocular tissues. The basis for lens-expression is similar for many, if not all, crystallins. The gene promoter contains response elements for some key transcription factors essential for normal lens development. Prominent among these are factors such as Pax6, c-Maf, Sox2, Prox1, and Six3 [93,94]. In many crystallin gene promoters, elements for lens and non-lens expression are combined in the same 5^′^ region. However, one of the more recently recruited enzyme crystallins, ζ-crystallin of the guinea pig (*C. porcellus*), illustrates dual promoter functionality quite nicely with an alternative promoter dependent on Pax6 and Maf elements, driving lens-specific expression [95].

## Conclusion

The human genome contains genes corresponding to all the major crystallins that are expressed in mouse and most other mammals. However, some of these (*CRYGEP*, *CRYGFP*, and *CRYGN*) are designated or likely pseudogenes in humans, while other genes have reduced expression compared with mouse. The genes for human crystallins encode proteins which belong to two unrelated superfamilies, the α-crystallin/sHSP superfamily and the βγ-crystallin superfamily (which in the human genome contains three genes for the AIM1 family). Ancestral members of these superfamilies were recruited to serve as crystallins in the earliest vertebrate lens, presumably because they could form and maintain the transparent, refractive medium needed to focus light. Humans also have another gene that has been recruited to a crystallin role more recently in evolution. The enzyme BHMT has very high expression in fetal lens where it may serve to replace those γ-crystallins whose expression has been reduced or eliminated. Indeed, crystallins have provided important early insights into the ways proteins can have multiple functions and can be adapted during evolution [4]. As might be expected, crystallins are implicated in many inherited cataracts, but in addition, due to their other roles as stress proteins or enzymes, proteins that serve as crystallins also show up unexpectedly in a number of disease states in eye and other tissues.

## Competing interests

The author has no competing interests.
